# Autologous micro-fragmented adipose tissue for the treatment of diabetic foot minor amputations: a randomized controlled single-center clinical trial (MiFrAADiF)

**DOI:** 10.1186/s13287-019-1328-4

**Published:** 2019-07-29

**Authors:** Roberto Lonardi, Nicola Leone, Stefano Gennai, Giulia Trevisi Borsari, Tea Covic, Roberto Silingardi

**Affiliations:** Department of Vascular Surgery, Ospedale Civile S. Agostino-Estense, Azienda Ospedaliero-Universitaria di Modena, University of Modena and Reggio Emilia, Via Giardini, 1355, 41126 Baggiovara, MO Italy

**Keywords:** Diabetes mellitus, Adipose tissue, Amputation, Peripheral arterial disease, Peripheral vascular diseases

## Abstract

**Background:**

The diabetic foot ulcer (DFU) is one of the most prevalent complications of diabetes mellitus and often develops severe effects that can lead to amputation. A non-healing “minor” amputation often precedes a major amputation resulting in a negative impact on the function and quality of life of the patients. Stem cell-based therapies have emerged as a promising option to improve healing, and the adipose tissue is an abundant and easy to access source. The injection of autologous micro-fragmented adipose tissue at the amputation stump of a diabetic population undergoing a lower limb minor amputation was evaluated and compared with the standard care.

**Methods:**

In this randomized controlled trial with two arms (parallel assignment) and no masking, 114 patients undergoing a lower limb minor amputation were randomized to standard of care or to micro-fragmented adipose tissue injection prepared using a minimal manipulation technique (Lipogems®) in a closed system. Clinical outcomes were determined monthly up to 6 months. Primary endpoint of the study was the evaluation of the healing rate and time after the minor amputation. Secondary endpoints included the assessment of safety, feasibility, technical success, relapse rate, skin tropism, and intensity of pain.

**Results:**

At 6 months, 80% of the micro-fragmented adipose tissue-treated feet healed and 20% failed as compared with the control group where 46% healed and 54% failed (*p* = 0.0064). No treatment-related adverse events nor relapses were documented, and technical success was achieved in all cases. The skin tropism was improved in the treatment group, and the pain scale did not differ between the two groups.

**Conclusion:**

The results of this randomized controlled trial suggest that the local injection of autologous micro-fragmented adipose tissue is a safe and valid therapeutic option able to improve healing rate following minor amputations of irreversible DFU. The technique overcomes several stem cell therapy-related criticisms and its potential in wound care should be better evaluated and the therapeutic indications could be expanded.

**Trial registration:**

ClinicalTrials.gov number: NCT03276312. Date of registration: September 8, 2017 (retrospectively registered).

## Introduction

Diabetes mellitus is a rapidly increasing chronic disease that has a significant impact on the communities’ health [1]. A common complication of this pathology is the development of chronic lower extremity ulcers, with the diabetic foot ulcer (DFU) being the most prevalent [2, 3]. A DFU often develops severe complications such as infection, which can lead to amputation and prolonged hospitalization. Every year, more than 1 million people are subjected to amputation due to DFU, and this number is underestimated taking into account the lack of a national registry in developing countries [1]. A non-healing digital or transmetatarsal “minor” amputation (DA; TMA) often precedes a major amputation resulting in a negative impact on the function and quality of life of the patients [4, 5]. A major amputation means physical disability and also psychological/psychiatric problems, with significant increase in the mortality rate, which is estimated to range from 10 to 50% and from 30 to 80% at 1 and 5 years post-amputation, respectively [6–9]. The DFU has been estimated to account for 12–15% of the overall financial resources destined to the management/treatment of diabetes [7–9]. The financial burden reverts on the patients, society, and the National Health System, and considering its not negligible amount, it becomes imperative to reduce the number of major amputations. That said, improving the stump healing following minor amputations seems to be reasonable and cost-effective.

Stem cell-based therapies have emerged as a promising therapeutic strategy to improve the healing process [10–13]. The attraction is additionally boosted by the absence of strong evidences able to demonstrate the superiority of any other specific conservative treatment or dressing [14]. Through trophic, immunomodulatory, and anti-microbial actions, mesenchymal stem cells (MSCs) “sense” and “signal” changes in the microenvironment where they reside by serving as paracrine mediators [11, 15, 16]. The adipose tissue is an abundant source of MSCs (ASCs), easy to access and simple to harvest [17, 18]. Both in vitro and in vivo studies showed favorable results and confirmed their anti-inflammatory and regenerative properties [11, 19–21]. To overcome the complex regulatory issues linked to the enzymatic treatment and/or cell expansion [22–25], a minimally manipulated autologous adipose tissue is a promising and safe option [26].

The commercially available Lipogems® system is a class II-a medical device intended for the closed-loop processing and transferring of autologous adipose tissue in a single surgical step. This technology reduces the size of the adipose tissue clusters by means of mild mechanical forces while eliminating pro-inflammatory oil and blood residue, intra-operatively providing mechanically micro-fragmented adipose tissue in a short time without expansion and/or enzymatic treatment [27]. Throughout the overall procedure, the processed fat is only subjected to slight mechanical forces without detrimental effects on the integrity of the stromal vascular niche and the tissue itself because the device is carefully prefilled with saline to avoid the presence of air throughout all the steps. The resulting product has been shown to possess reparative properties, particularly when injected into inflammatory or ischemic tissues [28] due to its capacity to induce vascular stabilization and to inhibit several macrophage functions involved in inflammation [29] and has been proven to have potential applications in osteoarthritis, anal incontinence, anal fistulas, low back pain, orthognathic surgical corrections, and others [30–39].

To the best of our knowledge, there are no randomized trials evaluating the injection of micro-fragmented adipose tissue at the amputation stump compared with the standard care. Therefore, a randomized controlled single-center clinical trial was performed in our department with the primary endpoint of assessing its impact in terms of healing rate and time in a diabetic population undergoing a lower limb minor amputation. Secondary endpoints included the safety, feasibility, technical success, relapse rate, skin tropism, and pain grading up to 6 months. It has to be highlighted that micro-fragmented adipose tissue has been extensively studied in other clinical areas and a number of published evidences showed no safety or feasibility concerns [30, 31, 33–35, 40].

## Materials and methods

### Study design and population

This is a randomized controlled single-center clinical trial (MiFrAADiF) with two arms (parallel assignment) and no masking. The trial has been performed (enrollment, treatment, clinical assessments, and result analyses) between 7 April 2015 and 31 March 2018 at the Diabetic Foot Service in the Vascular Surgery Department of the University of Modena and Reggio Emilia, Modena, Italy.

**Fig. 1:**
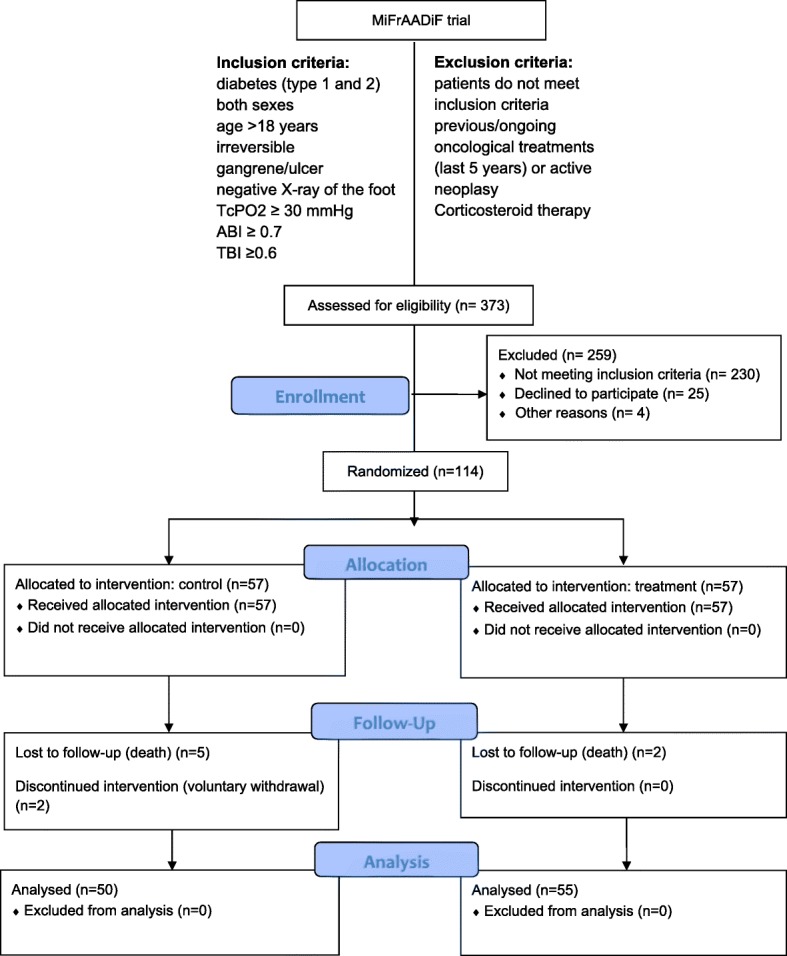
MiFrAADiF trial flow diagram, inclusion and exclusion criteria

Patients were selected according to the following inclusion criteria (Fig. 1): patients with diabetes mellitus (types 1 and 2) of both sexes, age > 18 years old, and presence of irreversible digital/forefoot ulcer/gangrene (with negative X-ray for osteolytic lesions). Adequate circulation (perfusion) was assessed by transcutaneous oxygen test ≥ 30 mmHg, ankle brachial index ≥ 0.7, and pressure index finger/arm toe/brachial index ≥ 0.6. Doppler arterial waveforms were triphasic or biphasic at the ankle of the affected leg. Patients who had undergone previous oncological treatments (past 5 years) or ongoing and/or neoplastic lesions, under corticosteroid therapy, with active vascular issues or inadequate lower extremity perfusion were excluded. Eligible patients were randomized 1:1 to local injection of autologous micro-fragmented adipose tissue (treatment group) or to standard clinical practice (control group) after a lower limb minor amputation.

### Randomization—sequence generation

Randomization used a paper block system. Sheets of paper in blocks of ten with five sheets having an assignment of treatment and the other five having the assignment of control were placed in a blank sealed envelope. The envelopes were shuffled and then labeled 1 through 10. This process was observed by the principal investigator and study staff. The investigators did not have knowledge of the process used to create the assignments, and randomization of patients proceeded individually at their first post-screening treatment.

### Surgical procedure

The care of all the patients as well as the amputations was carried out in accordance with the international standards [7]. All procedures were performed in an operating theater. Local or regional anesthesia and eventual superficial sedation were performed by the anesthesiologist. Patients from both arms were subjected to DA or TMA minor amputation. The stumps were closed by primary intention. After amputation, patients were treated as follows:

A) Treatment group. In the same surgical session, the lower/lateral abdomen or the inner/outer thigh was chosen as the donor site. Both donor and distal site were cleaned with chlorhexidine. Prior to harvest, the donor site was injected with 100 mL of Klein Solution (500 mL saline, 1 mL epinephrine 1/1000 IU, and 40 mL lidocaine 2%) using a disposable 17-gauge cannula connected to a 60-mL Luer lock syringe. The fat was then harvested (50–100 mL) using a 13-gauge cannula connected to a 20-ml VacLok® syringe. The lipoaspirated tissue was immediately processed in the Lipogems® processing kit (Lipogems International Spa, Milan, Italy) as previously described [27].
  Lipogems® is a disposable device that mechanically reduces the size of the adipose tissue clusters while eliminating oily substances and blood residues in a complete immersion of physiological solution to minimize any mechanical-related trauma on the cells. The device consists of a cylindric processing unit which contains five stainless steel marbles, an input and an output sieve, a saline input line, an access port with Luer lock connection to load the lipoaspirate, a drainage line, a second access port with Luer-lock connection to unload the processed material, and a collection bag for waste fluid. The processing unit, filled with normal saline solution is maintained in flow condition by gravity. After saline priming, adipose tissue inserted in the device undergoes a first cluster reduction and micronization by means of the input sieve. The mechanical action exerted by shaking the stainless steel marbles in the processing unit allows emulsion of the lipid mass and consequently the reduction of the clusters. The continuous flow of normal saline solution eliminates residues of oil emulsion and any remaining blood components. The second adipose cluster reduction is obtained by passing the floating adipose clusters through a second-size reduction filter. At the end of the procedure, the device releases a micro-fragmented fluid fat tissue product (clusters of 300–600 μm in diameter) that can easily pass through a small caliber needle. The processed micro-fragmented fat was collected in a 60-mL syringe to decant, and the excess of saline solution was eliminated. The final product was injected radially into the bed of the amputation in an amount dependent on the extension of the stump within a range of 10 to 30 mL. The entire process was carried out in sterile conditions into the operating theater.
  The medication was carried out after cleaning with sodium hypochlorite and saline solution, using paraffin gauze with a povidone-iodine solution (10% of iodine). Compressive medication was applied to the site of fat harvesting for the following 48 h.
  Patients were kept under observation and discharged from the hospital with the instruction of absolute rest and unloading on the limb until the next control.
B) Control group. The medication was carried out after cleaning with sodium hypochlorite and saline solution, using paraffin gauze with a povidone-iodine solution (10% of iodine).
  Patients were kept under observation and discharged from the hospital with the instruction of absolute rest and unloading on the limb until the next control.

### Follow-up, outcomes, and definitions

All the patients were clinically assessed within 20 days from surgery and monthly for 6 months thereafter.

At each visit (enrollment and follow-up), the patients were clinically evaluated [7] and asked to grade pain through the visual analogue scale (VAS). All the visits were performed by a selected group of experienced physicians (RL, SG, RS) and dedicated investigators (NL, GTB, TC) who simultaneously gathered the data on a case report form (paper and electronic).

The DAs (finger or trans-phalangeal) and the TMAs (up to midfoot) were considered minor amputations.

The primary objective of the MiFrAADiF trial was the evaluation of healing rate and time of the minor amputations treated with autologous micro-fragmented adipose tissue compared with standard of care in case of irreversible DFU with resolved peripheral arterial disease.

Healing was defined as complete re-epithelialization of the stump by primary intention, as determined by at least 2 investigators. A visit was conducted 7–10 days after healing to confirm the result. Failure was considered a stump dehiscence requiring any kind of foot re-operation (revision, secondary minor or major amputation), infected or non-healing amputation at the end of the follow-up.

Secondary outcomes included the assessment of the safety, the feasibility, the technical success, the relapse rate, the skin tropism, and the intensity of pain. Safety was assessed by recording type and incidence of any adverse event and complications occurring during the follow-up, discriminating the likely relationship between the complication and the technique. Feasibility was intended as any technical issue encountered in adipose tissue harvesting, processing, and injection in the amputation stump. The technical success was considered achieved in case of absence of issues precluding the procedure. Relapse was defined as clinically assessed stump re-dehiscence after a “false” healing. The skin tropism of the stump was assessed by a combined evaluation of the perilesional skin (graded as undamaged, erythematous, macerated, atrophic) and of the lesion’s edge (graded as undamaged, erythematous, callous, macerated, necrotic). All these items were graded (0 cm, 1 cm, 1.5 cm, > 2 cm), and the sum was used to evaluate the tropism of the skin. Pain was assessed with the VAS.

### Sample size calculation and statistical methods

The correct sample size was calculated considering as primary endpoint a benefit in terms of a 50% reduction in the healing time. The mean healing time for a lesion is generally 4 months; thus, a reduction to 2 months can be considered a clinically relevant benefit. Considering a dropout of 10% by setting a level of significance alpha = 0.05 and a power of 1 − beta = 0.80, 57 patients for each arm were required.

The primary endpoint, i.e., the difference in the healing time between the two groups, has been calculated by log rank test and Kaplan-Meier curves. Secondary outcomes have been analyzed by appropriate one-way ANOVA with Bonferroni’s post-test for continuous variables after normality assessment by Kolmogorov-Smirnoff test. To test the influence of multiple variables on continuous data, two-way ANOVA has been performed with Bonferroni’s post-test. For categorical variables, Fisher’s exact test was applied. All these analyses were performed using GraphPad Prism v7.0 (GraphPad Software, La Jolla, CA, USA).

A multivariate logistic regression analysis was applied to identify influencing factors, using R software (R Foundation, Vienna, Austria). A *p* value < 0.05 was considered statistically significant and a *p* < 0.1 was considered as tendency.

### Ethics

The MiFrAADiF trial was conducted under Local Ethics Committee approval (protocol no. 2621/C.E.). The written consent and the case report form were reviewed by the Local Ethics Committee. The participants’ written consent was obtained prior to the enrollment. The trial was registered in ClinicalTrials.gov (NCT03276312) and conducted in compliance with applicable regulatory requirements in accordance with the revised provisions of the Helsinki Declaration and in adherence with Good Clinical Practice. Confidentiality was maintained with all patient records.

## Results

A total of 373 subjects were screened, and the 114 meeting the inclusion criteria were enrolled and randomized 1:1 to micro-fragmented adipose tissue injection (treatment group, *n* = 57) or standard of care (control group, *n* = 57) (Fig. 1). Patients and amputations’ level baseline characteristics were similar (Table 1).

**Table 1:** Background data of the population

	Treatment group (*n* = 57)	Control group (*n* = 57)
Age (years old)		
Mean	69.0	71.6
Standard deviation	11.6	10.8
Gender		
Male	45 (79%)	41 (72%)
Female	12 (21%)	16 (28%)
First treatment		
Yes	49 (86%)	53 (93%)
No	8 (14%)	4 (7%)
Type of amputation		
Digital	49 (86%)	49 (86%)
Transmetatarsal	8 (14%)	8 (14%)
Related pathologies	53 (93%)	57 (100%)
Hypertension	50 (88%)	50 (88%)
Chronic renal failure	20 (35%)	28 (49%)
Hemodialysis	6 (10%)	1 (2%)
Heart diseases	35 (61%)	43 (75%)
Neurological disorders	2 (4%)	8 (14%)
Autoimmune disorders	1 (2%)	–
Chronic respiratory failure	9 (16%)	14 (25%)
Others	9 (16%)	10 (18%)
Smoke		
Yes	11 (20%)	8 (14%)
Former	23 (40%)	24 (42%)
No	23 (40%)	25 (44%)
Concomitant therapies	57 (100%)	57 (100%)
Oral anticoagulant	8 (14%)	15 (26%)
Antiplatelet	53 (93%)	53 (93%)
Insulin	37 (65%)	46 (81%)
OHAs	32 (56%)	25 (44%)
NSAIDs	8 (14%)	9 (16%)
Opioid	6 (10%)	7 (12%)
Antibiotics	10 (18%)	6 (10%)
Others	7 (12%)	3 (5%)

Continuous data are presented as means and standard deviation. Categorical data are given as counts (%)

*OHAs* oral hypoglycemic agents, *NSAIDs* non-steroidal anti-inflammatory drugs

At 6 months, 80% (*n* = 44/55) of the micro-fragmented adipose tissue-treated feet healed and 20% (*n* = 11/55) failed as compared with the control group where 46% (*n* = 23/50) healed and 54% (*n* = 27/50) failed (*p* = 0.0064 treatment vs. control).

Apart from micro-fragmented adipose tissue, also concomitant therapies such as oral hypoglycemic agents (*p* < 0.05) and antibiotics (*p* < 0.05) positively correlated with healing. On the other hand, the presence of cardiac pathologies, chronic respiratory insufficiency, hemodialysis, and age were found as negative factors but only for the treatment group (*p* < 0.05, *p* < 0.05, *p* < 0.05, and *p* = 0.08, respectively).

**Fig. 2:**
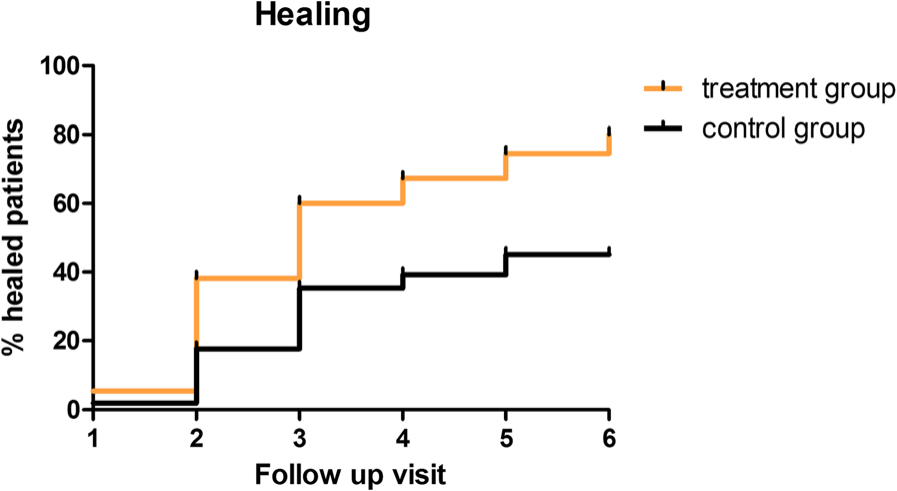
Kaplan-Meier showing healing probability over time

The healing time did not differ between the 2 groups, resulting in an average of 2.8 months (SD 1.3 months) in the treatment group and 2.8 months (SD 1.0 month) in the control group. On the contrary, the healing probability during time was significantly improved in the treatment group with respect to the control group (*p* < 0.001, Fig. 2). In addition, patients with a digital lesion appeared to recover faster (within the fourth follow-up visit; *p* = 0.034).

As shown in Fig. 1, seven deaths not treatment-related were documented during this trial (2 in the treatment group and 5 in the control group). In addition, two patients from the control group discontinued intervention due to voluntary withdrawal. Two procedure-related complications were registered (2 hematoma of the abdominal wall, site of adipose tissue harvesting); one was resolved with compressive dressing, and the second required a surgical incision to achieve hemostasis of the subcutaneous tissue. Both patients were prior assuming oral anticoagulant medication.

Technical success was achieved in all cases, and no technical issues precluded the completion of the procedure. The extremely fluid fat easily passed through fine sharp needles (21 up to 25 G) and was distributed uniformly in the bed of the amputation.

No relapses have been observed in both study groups.

Regarding skin tropism, no correlation between perilesional skin or lesion’s edge at baseline and healing or failure has been detected. The skin tropism appeared to be improved in the treatment group because healing improvement was achieved in a higher number of patients as compared to control.

**Fig. 3:**
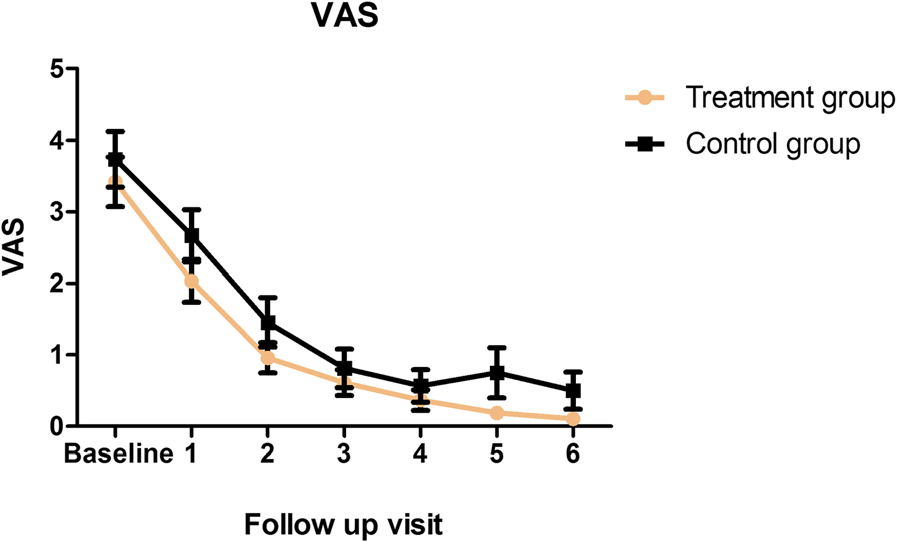
Kaplan-Meier showing visual analogue scale (VAS) over time

The VAS scale did not differ between the groups at any follow-up visit (Fig. 3). The improvement of this parameter was registered in both groups since the second visit with respect to the preoperative values. The pain reduction was mainly influenced by the time (*p* < 0.001), and the treatment with micro-fragmented adipose tissue demonstrated a significant contribution to this effect (*p* < 0.05).

## Discussion

The healing of the amputations performed in case of diabetic foot ulcers (DFU), both digital (DA) and transmetatarsal “minor” amputation (TMA), represents a challenge. The high burden of the disease [1], meaning millions of people losing quality of life, and the elevated costs to health providers [9] are the leading factors pushing medical research.

The present randomized controlled trial compared the injection of autologous micro-fragmented adipose tissue (treatment group) at the amputation site compared with standard care primarily aiming at improving the healing rate in such arduous clinical setting. The presence of mesenchymal stem cells within the adipose tissue (ASCs) and the promising results documented in the treatment of different pathologies [30–35] prompted our group to evaluate the efficacy of this innovative technique in improving stump healing after a lower limb minor amputation. The pathophysiology of the DFU includes microcirculatory damages and growth factor alterations, plus other mechanisms that keep the wound in an inflammatory phase [41]. The beneficial role of ASCs has been demonstrated by many authors [18–20, 42, 43]. The multi-potent differentiation capability together with the strong paracrine action of these cells represent an interesting therapeutic chance to increase healing [10–13] and to treat the commonly underlying arterial disease.

The technique we selected in this trial requires lipoaspiration, which has been used for decades in plastic surgery with a very low incidence of major complications [44]. The absence of procedure-related death and major adverse events allowed us to confirm the safety of both the lipoaspiration and the injection of the autologous micro-fragmented adipose tissue in the amputation stump.

The present study provided a significantly higher healing rate of the treatment group compared with the control (80% vs. 46%, *p* = 0.0064) which was totally unexpected considering a recent meta-analysis [5] that reported a re-amputation rate exceeding 55% following TMA. Our results were obtained excluding from the study second intention healing and healing after 6 months, two common resolution of minor amputations. Moreover, not all the patients analyzed in the meta-analysis were diabetics and the inclusion criteria were not precise [5]. In the present authors’ opinion, all of these aspects make the results of MiFrAADiF study even more interesting.

A clinical evaluation of the skin tropism was performed at each follow-up visit, but this outcome can be affected by plenty of confounding factors and several considerations have to be made. A unique exam testing the skin tropism validated by the scientific community is not available. The standardization of a clinical evaluation is still difficult in spite of the fact that these assessments were performed by a selected team of experienced physicians being part of our Diabetic foot service using a standard case report form. The skin tropism appeared to be improved in the treatment group just because healing improvement was achieved with such a big difference compared to control.

Pain assessment represented another secondary outcome of the present study. No significant differences between the 2 groups were found at each follow-up visit using the VAS pain scale. Anyway, the treatment group had a reduction of pain in a significant shorter time. The no-difference in pain grading could be of no surprise if we consider that the high prevalence of diabetics suffer symptoms of distal polyneuropathy [45]. The pathophysiology of this phenomenon and the correlation with DFU is not yet completely understood [45]. Specific publications about pain after minor amputations for DFU are not available, partially due to the use of health-related quality of life tools. These tools combine physical, mental, and social health data including pain grading. It is difficult to discern specific amputation related from the neuropathic pain in such complex patients, especially in the “acute” phase of the amputation. Furthermore, lifestyle and pharmacological interventions are not able to treat completely neuropathic pain and its evolution has been defined “not predictable” in a recent review [45]. The intra-articular injection of autologous micro-fragmented adipose tissue in osteoarthritic patients has been demonstrated to significantly improve the VAS scale [40]. It is the authors’ opinion that these findings are encouraging and probably our not statistically significant results were biased by the overlapping neuropathy and the well-known confounding factors affecting the DFU.

The major re-amputation rate was reported to range from 0 to 56% in a review focused on TMA (including non-diabetic patients) [5], and the rate reached 63% considering all the re-operations. Published data on TMA outcomes led some authors to doubt the primary TMA approach in lieu of minor amputations [5]. The failure rate in the present trial (re-amputations, re-operations, not-healing stump at final follow-up) was 20% in the treatment and 54% in the control group. In addition, the oral hypoglycemic agents and antibiotics were found to be correlated. The prevalence of type II diabetic patients in this trial led us to hypothesize that patients taking insulin had an overall worse disease compared with OHAs patients and tent toward worse results. Obviously, this is a speculation and future studies are mandatory.

To conclude, the DFU is a tough clinical and medical field. The MiFrAADiF trial demonstrated the extremely high benefit of the injection of autologous micro-fragmented adipose tissue in terms of healing. This is a partial answer to the continuous demand of randomized controlled trials focused on regenerative therapies that still dominate the wound healing scientific community.

Several factors were taken into account when planning the trial with micro-fragmented adipose tissue: the harvesting is safe and simple [44], the mechanical fragmentation avoids laboratory manipulation of the product (e.g., enzymatic treatment), the use of minimally manipulated autologous adipose tissue complies with ethics laws, the immunological rejection could be avoided without heterologous and/or allogeneic material, and injecting the graft at the stump level avoids possible complications related to the endovascular delivery [13]. The results lead us to confirm the abovementioned positive features, in particular, the intra/perilesional injection was safe and feasible in a wide range of stumps (DA/TMA). Because the trial focused on the feasibility and efficacy of a product obtained with a commercially available device, a fine morphological analysis of the injected material was not within the aims of the study. Furthermore, the injected micro-fragmented adipose tissue has been widely studied and characterized in vitro by other authors [27, 29, 46–48] and published data indicate that it contains an abundant number of cells able to act through a paracrine mechanism to prime and sustain angiogenic, anti-fibrotic, anti-inflammatory, and immunomodulatory responses in the target tissue. The number of injected cells was not assessed because it is a hard technical issue, due to the presence of cell aggregates upon collagenase digestion that prevent an exact cell count. It should be highlighted however that micro-fragmented adipose tissue is a complex matrix that contains not only MSCs but also many other active elements embedded in a natural scaffold that preserves them from a rapid degradation in vivo. Thus, the number of injected MSCs cannot be simplistically assumed as a measurement of efficiency. Application of micro-fragmented adipose tissue in experimental animal ischemic disease models has shown some beneficial effects mediated by the capability of its MSC content to release vasculogenic/angiogenic and anti-inflammatory molecules [28, 29]. An in vitro study of its angiogenic activity demonstrated that it significantly reduces adhesion molecule (AM) expression (ICAM-1 and VCAM-1) and improves cord-like formation, indicating a preferential ability to favor vascular stability and maturation.

The analysis of the secretome indicated a high content of both angiopoietin-1 and angiopoietin-2 and low levels of vascular endothelial growth factor (VEGF) and matrix metalloproteinase-2 (MMP2). In addition, the cultured micro-fragmented adipose tissue releases in its culture medium a number of anti-inflammatory factors. Indeed, it reduced migration, adhesion to an activated endothelial cell monolayer, and the release of Regulated upon activation normal T cell expressed and presumably secreted (RANTES) and monocyte chemotactic protein-1 (MCP-1) chemokines of U937, monocytes of tumorigenic origin used as a valid model to investigate the inflammatory properties of monocytes. These data indicate that micro-fragmented adipose tissue in vitro is able to block several important monocyte inflammatory functions [28, 49].

It must be taken into account that the impairment of the angiogenic/differentiation potential of the autologous stem cells in diabetes still represents an issue to be investigated [50].

All that said, the commercially available Lipogems® system has been demonstrated to be easy-to-use (no technical failure), quick (short procedural time), and versatile (no specific characteristics in the patient are required). The kit-related costs seem to be inferior to more complex stem cell manipulation systems. Regardless of the initial cost, future publications should evaluate the hypothesis of a reduction of the global economic burden due to the application of autologous micro-fragmented adipose tissue in the treatment of DFU.

The present study is not without limitations due to the absence of blinding which represents the most important critical issue. Unfortunately, the “waste” harvesting of adipose tissue is not ethically acceptable because of its invasiveness. In addition, the funding constraints and the volume of the center forced us to limit the study sample size. Although the sample size calculation indicated that the number of patients we enrolled and treated was sufficient, a higher number of patients in a multicenter setting would have been preferable from a clinical point of view. The number of MSCs was not assessed in order to evaluate the “quality” of the micro-fragmented adipose tissue of each patient, and to the best author knowledge, the optimal amount of stem cells to inject is not yet defined [50].

Future work should clarify if the impressive healing results reported above correlate with a reduction of the hospitalization time and of the overall health-related costs. These perspectives could have an even more positive impact on patients’ quality of life and on the health providers’ policy. Further points to be addressed should be the impact of the technique on pain, encompassing pathophysiological study to understand the overlap with neurological disorders.

## Conclusions

The single-center, prospective, controlled, and randomized design represents the strengths of the present work and the gold standard in order to minimize investigator, selection, and information bias as well as to manage confounding factors.

Our experience with autologous micro-fragmented adipose tissue has proven to be a valid therapeutic option able to drastically improve healing following minor amputations performed on DFU. The present technique overcomes several stem cell therapy-related criticism and its potential in wound care should be better evaluated and the therapeutic indications could be expanded.

## Data Availability

All the data of the current trial are available from the corresponding author on reasonable request.

## Abbreviations

ASCs: Adipose tissue mesenchymal stem cells
DA: Digital amputation
DFU: Diabetic foot ulcer
MiFrAADiF: Micro-fragmented autologous adipose tissue for diabetic foot
MSCs: Mesenchymal stem cells
TMA: Transmetatarsal amputation
VAS: Visual analogue scale
